# Reduced-Cost Production of Sophorolipids by *Starmerella bombicola* CGMCC1576 Grown on Cottonseed Molasses and Cottonseed Oil-Based Medium

**DOI:** 10.3390/ijms24065759

**Published:** 2023-03-17

**Authors:** Zehua Qin, Wei Guo, Jun Liu, Guoqin Zhao, Mingxin Liu, Xin Song

**Affiliations:** 1 State Key Laboratory of Microbial Technology, Shandong University, Binhai Road 72, Qingdao 266237, China; 2 National Glycoengineering Research Center, Shandong University, Binhai Road 72, Qingdao 266237, China

**Keywords:** *Starmerella bombicola*, sophorolipids, cottonseed molasses, cottonseed oil, response surface optimization

## Abstract

A large-scale application of sophorolipids (SLs) was blocked by their high production cost. One feasible way to reduce the cost of SL production is to develop cheap feedstocks as the substrates for SL fermentation. In the present work, cottonseed molasses (CM), a waste from raffinose production, was used as the hydrophilic substrate;, and cottonseed oil (CO) was used as a hydrophobic substrate for SL production by *Starmerella bombicola* CGMCC 1576. The primary optimization of carbon sources, nitrogen source and inorganic salts, produced 57.6 ± 2.3 g/L of total SLs and 24.0 ± 1.2 g/L of lactonic SLs on CM and CO, almost equal to the titer of SLs produced from glucose and oleic. A response surface method was applied to optimize the fermentation medium for growth and SL production of *S. bombicola*. The production of total SLs reached 58.4 ± 3.4 g/L, and lactonic SLs were elevated to more than 25.0 ± 1.9 g/L. HPLC–MS analysis showed that the compositions of SLs produced by *S. bombicola* on CM and CO were very similar to those on glucose and oleic acid. These results suggested that cottonseed molasses and cottonseed oil can be used as renewable cheap substrates for the reduced-cost production of SLs.

## 1. Introduction

The demand for surfactants in a variety of industrial sectors, such as pharmaceutical, cosmetics, food processing, detergent, petroleum, and environment industries, has been increasing. However, the use of chemically synthesized surfactants may lead to significant ecological problems, particularly in washing applications as these surfactants inevitably end up in the environment after use [1]. Hence, biosurfactants, produced by microorganisms, including bacteria, yeast, and mold, have attracted much more attention due to their good surface activity, biodegradability, biocompatibility, low toxicity, production under mild conditions, and from renewable materials [2]. Sophorolipids (SLs) are mainly produced by *Candida apicola*, *Candida bogoriensis*, *Candida bombicola*, *Wickerhamiella domercqiae*, *Pichia anomala*, or *Rhodotorula bogoriensis* and have the highest yield of all biosurfactants [3]. Among them, the yeast *Starmerella bombicola* CGMCC 1576, a sophorolipid-producing non-pathogenic yeast, was isolated from oil-containing wastewater and initially identified as *Wickerhamiella domercqiae var. Sophorolipid* was based on physiological and biochemical identification tests and was later reclassified as *Starmerella bombicola* based on sequence analysis [4]. SLs are produced as a mixture of more than 20 different sophorolipid molecules. All of these different SL molecules have similar basic structures, that is, a hydrophilic moiety, one dimeric sugar sophorose molecule linked by a glycosidic bond to a hydroxyl group on a long-chain fatty acid - the lipophilic part. Some small modifications that occurred on the basic chemical structures produce different SL molecules. SLs produced by the above yeasts were a mixture of different SL molecules, which vary in hydroxy fatty acid with different carbon chain lengths and saturation degrees, the acetylation position, the number of acetyl groups on the sophorose head, and the position of hydroxyl group on the lipophilic fatty acid tail [5].

In spite of the highest yield of SLs among all biosurfactants, the production cost of SLs, which is three-to-six times higher than that of chemical surfactants, has still hindered the large-scale production and application of SLs. Two traditional strategies were adopted to reduce the production cost of SLs, one of which is to improve the production condition and process. The downstream processes account for about 60% of the total cost of SLs production [6]; therefore, they are still the main technological limitation for industrial exploitation [7]. Hence, many efforts on the process optimization of SL production have been made. The highest concentration of SLs was produced by the cultivation of *C. bombicola* in a two-stage cultivation process [8]. A fed-batch combined two-stage continuous process [9] and feeding rate-controlled fed-batch process [10] were performed to produce SLs. Semicontinuous SL fermentation was also performed by using a novel bioreactor with dual ventilation pipes and dual sieve-plates, coupled with a novel separation system [3]. Recently, Banat et al. presented solid-state fermentation as a production technology for SLs [11]. Many investigations have highlighted that biosurfactants are produced in higher quantities using Solid-State Fermentation compared to liquid fermentation and the same culture conditions, e.g., temperature, nutrient concentration, and agitation. The product yield by *P. ostraeus*, for example, was two-fold higher when produced in Solid-State Fermentation than in liquid fermentation.

Given that the cost of feedstock account for approximately 30% of the overall cost [12], another way to reduce production costs is to develop cheap and renewable industrial and agricultural wastes as alternative substrates for SL production. Over the past decades, researchers have been trying to use a variety of industrial and agricultural wastes as hydrophilic and hydrophobic substrates for SL production. Deproteinized whey concentrate was first used as the substrate for the production of SLs [13]. Since then, molasses, including sugarcane and soybean molasses, is considered to be a suitable and cheap substrate to replace glucose. Molasses consists mainly of sugars, including sucrose, oligosaccharides, and a small amount of monosaccharides. Sucrose molasses and three different oils were used as carbon sources for the fermentation of SLs and when sucrose molasses and soybean oil were used as carbon sources, SLs produced the highest titer of 23.3 ± 1.6 g/L, which was equivalent to glucose medium and greatly reduced the production cost [14]. By using soybean molasses and oleic acid as substrates, the yield of SLs was 53.0 ± 2.8 g/L, which reached about 70% of that produced from glucose medium [15]. In addition, researchers have also used deproteinized whey concentrate [13], sweetwater [12], etc., to replace hydrophilic carbon sources (glucose) to produce SLs. Ma et al. also used the waste residue from a delignified corncob to produce SLs, and the production of SLs could reach 49.0 ± 5.7 g/L [4]. In addition, a variety of hydrophobic materials, such as biodiesel by-product streams, soybean black oil, waste frying oil, industrial fatty acid residues, restaurant waste oil, and soybean dark oil, have been used to replace common hydrophobic carbon sources (vegetable oils and alkanes, etc.) for the synthesis of SLs [16,17,18,19,20]. Three rare hydrophobic substrates, tapis oil, melita oil, and ratawi oil, were used for the production of SLs, and the SL yields reached 26 ± 0.7 g/L, 21 ± 2.4 g/L, and 19 ± 1.9 g/L, respectively [21]. As for the nitrogen source in the medium (such as yeast extract), it can be substituted by corn steep liquor, peptones, and other nitrogenous substances [22]. These various raw materials were used as the hydrophilic and hydrophobic subtrates for SL production, mainly at laboratory levels and because of the difficulty of the collecting of raw materials (restaurant waste oil, industrial fatty acid residues), troublesome processing procedures of the raw materials (delignified corncob residue), small quantities, and large volume (sweet water).

China is the world’s top producer of cotton, with an annual production of about 7-million tons, and cotton seeds account for 35–40% of the total weight of cotton. Cottonseed molasses and cottonseed oil were both derived from cottonseed. Cottonseed molasses was a waste material to be discarded in the production of raffinose; it is not well utilized. Cottonseed oil made by cottonseed pressing is difficult to use as an edible oil because it is difficult to remove gossypol. However, so far, there have been no reports on the production of SLs using cottonseed molasses and cottonseed oil as cheap substrates. Hence, they are expected to be turned from waste as the substrates for SL production. In this study, we aimed to explore the feasibility of using cottonseed molasses and cottonseed oil as substitutes of glucose and oleic acid in the fermentation medium for SL production and expected to optimize the fermentation medium containing cottonseed molasses and cottonseed oil by statistical methods to reduce the production cost of SLs.

## 2. Results and Discussion

### 2.1. Composition Analysis of Cottonseed Molasses and Cottonseed Oil

By using cheaper renewable industrial and agricultural wastes as the substrates, the production cost of SLs is expected to be greatly reduced and, thus, become cost competitive with chemically synthesized surfactants. In the past decades, researchers have been trying to use a variety of industrial and agricultural wastes as hydrophilic and hydrophobic substrates for SL production. Previous research found that soybean molasses, instead of glucose as the hydrophilic carbon source, and costly yeast extract and urea as the nitrogen sources, were developed to produce SLs from *Candida bombicola*. A product yield achieved 71 ± 4% g of purified SLs per liter of starting culture volume [23]. However, the production cost of SLs from the above ferementation medium formula is somewhat expensive. As the by-product of raffinose production, the cost of cottonseed molasses is lower than that of soy molasses. The composition analysis by HPLC (Figure S1) also showed that cottonseed molasses contains a large amount of soluble sugar and protein. The composition and content of cottonseed molasses (*w*/*v*) are as follows: raffinose 21.1%, xylose 0.6%, stachyose 4.6%, melibiose 1.4%, sucrose 25.9%, fructose 0.7%, glucose 1.4%, oligosaccharides 5.2%, crude protein 20.2%, and water 19.0%. The HPLC results showed that, besides some oligosaccharides and trisaccharide raffinose, cottonseed molasses is mainly composed of a relatively high content of sucrose, some disaccharides, and monosaccharides. In addition, it contains a certain amount of protein and inorganic salts (). The advantage of cottonseed molasses is that it not only provides the required carbon source but also protein and inorganic salts for yeast growth. Therefore, cottonseed molasses is expected to be a better fermentation substrate for SLs production.

Compared with soybean oil and rapeseed oil, the production cost of SLs from biodiesel by-product flow (BCS) and frying waste oil can be reduced [1]. However, difficulties in the collection and separation of small quantities may be major obstacles to BCS and frying waste oil as the substrate for the production of SLs with an annual production of about 7-million tons of cotton in China and cotton seeds account for 35–40% of the total weight of cotton so that the quantities of cottonseed oil are huge because cottonseed contains more than 35% cottonseed oil. Hence, cottonseed oil has the advantages of large quantity and low price. Cottonseed oil is expected to be a better and cheaper fermentation substrate for SL production and the reduction in production cost, which is very likely to promote the industrial production of SLs. The chemical composition of the three hydrophobic carbon sources, oleic acid, soybean oil, and cottonseed oil, was used in this study. These three hydrophobic carbon sources were analyzed by gas-phase chromatography. The element was shown in Table 1. It could be found that the compositions of cottonseed oil and soybean oil were similar. Octadecanoic acids, including oleic acid and linoleic acid, account for a high significant proportion in cottondeed oil. Soybean oil was often used as a hydrophobic substrate for SL fermentation.

Cottonseed oil is similar in composition to soybean oil C16 and C18 concentrations in cottonseed oil reach 99%, but because cottonseed oil contains more palmitic acid, the composition of SL mixture prepared by cottonseed oil may be slightly different from that prepared by soybean oil as a hydrophobic substance. Under guaranteed fermentation yields, cottonseed oil is expected to be a better fermentation substrate.

### 2.2. SLs Production Using Cottonseed Molasses and Cottonseed Oil

The results of fermentations by using different hydrophilic carbon sources and hydrophobic carbon sources were shown in Table 2. When glucose and cottonseed oil were used as the hydrophilic substrate, *S. bombicola* produced the highest titer of total SLs (72.4 ± 2.3 g/L) and lactonic SLs (24.5 ± 1.8 g/L), which increased by 29.7% and 16.1%, compared with SL production from the medium containing glucose and oleic acid. When *S. bombicola* used cottonseed molasses and cottonseed oil as the substrates, the production of SLs reached a high level (68.9 ± 4.9 g/L), which was slightly lower than that produced from glucose and cottonseed oil. Compared with that produced in glucose medium, the biomass of *S. bombicola* grown in cottonseed molasses medium was significantly increased. The above results indicated that the production of total SLs and lactonic SLs are significantly increased by cheap cottonseed molasses and cottonseed oil when compared with other cheap substrates.

### 2.3. Production of SLs from the Medium Containing Different Concentrations of Yeast Extract and Cottonseed Molasses

The concentration of ammonium sulfate concentration greatly influences the growth and SL production of *S. Bombicola,* and it was concluded that a high nitrogen concentration was suitable for the growth of yeast but not for the synthesis of SLs [23]. The higher biomass produced from cottonseed molasses medium probably suggested that cottonseed molasses contains a high content of crude protein, which can be used as a nitrogen source for the fermentation of *S. bombicola*. The reduced SL production from cottonseed molasses than from glucose might be due to the high content of crude protein in cottonseed molasses, which could be used as the nitrogen source for *S. bombicola* growth and SLs production. Therefore, the yeast extract added to the cottonseed molasses medium should be less than that added to the glucose medium to prevent excessive nitrogen from adversely affecting the SL synthesis of *S. bombicola*. Different concentrations of yeast extract were added to the fermentation medium containing cottonseed molasses, respectively. The results were shown in Table 3. When the carbon sources were cottonseed molasses and cottonseed oil, with the increase of the amount of yeast extract, the biomass of *S. bombicola* increased, the production of both total and lactonic SLs significantly decreased, and the results confirmed the above speculation that cottonseed molasses contains sufficient nitrogen for *S. bombicola* to grow and does not require the addition of yeast extract. Compared with 50.0 ± 3.7 g/L total SLs and 28.2 ± 1.7 g/L lactonic SLs produced in the glucose and oleic acid medium, the total and lactonic SLs reached 49.2 ± 2.1 g/L and 27.6 ± 2.4 g/L in the fermentation medium containing CM and CO. Thus, it is unnecessary to add yeast extract to the culture medium with cottonseed molasses and cottonseed oil as the hydrophilic and hydrophobic carbon sources. The reduced cost production was achieved by simplifying the medium formulation.

### 2.4. Effects of Addition of Inorganic Salts to the Medium Containing Cottonseed Molasses and Cottonseed Oil on SLs Production and the Biomass of S. bombicola

The strain *S. bombicola* was cultured in a medium containing only cottonseed molasses and cottonseed oil. Inorganic salts were added to this medium as the control. The results were shown in Table 4. Whether inorganic salts were added or not to the molasses medium did not affect the final yield of SLs. The final titer of SLs in cottonseed molasses medium with and without inorganic ions was almost the same, and the biomass of medium without inorganic salts was slightly higher. The inorganic salts in cottonseed molasses are sufficient for *S. bombicola* to grow and produce SLs. Therefore, the cottonseed molasses medium does not need to add inorganic salts, further simplifying the fermentation medium.

### 2.5. Concentration Optimization of CM and CO by Single-Factor Gradient Experiment

According to the above experimental results, cheap medium only contained cottonseed molasses and cottonseed oil. The concentration of cottonseed molasses was optimized firstly. The concentration of cottonseed oil was set at 6.0% (*w*/*v*), and then different concentrations of cottonseed molasses were used as hydrophilic substrates for SL fermentation, respectively. The common medium containing glucose and oleic acid was used as the control. As shown in Table S1, as the concentration of cottonseed molasses increased, the biomass and the production of SLs both increased. When the content of cottonseed molasses was 11.7%, the production of both total and lactonic SLs reached the maximum, 48.9 ± 1.3 g/L and 24.5 ± 1.1 g/L, respectively, and the biomass was the lowest, 12.3 ± 0.6 g/L. When the amount of cottonseed molasses was higher than 11.7%, the biomass continued to increase but the production of total and lactonic SLs showed a downward trend simultaneously. When the concentration of cottonseed molasses increased to 15.0%, the biomass increased significantly, while the production of SLs decreased significantly. The content of 11.7% of cottonseed molasses in fermentation medium can be an optimized concentration for the subsequent concentration optimization experiments of cottonseed oil. The concentration optimization results of cottonseed oil were shown in Table S2. It can be seen that *S. bombicola* could grow well with little changes in the biomass under different concentrations of cottonseed oil. When the concentration of cottonseed oil was 7% (*v*/*v*), the production of both total and lactonic SLs reached the highest, 50.6 ± 1.8 g/L and 27.7 ± 0.3 g/L, respectively. The biomass was also the highest, 8.9 ± 0.5 g/L. When the concentration of cottonseed oil was higher or lower than 7%, the production of SLs was lower than that at 7% of cottonseed oil. When 11.7% cottonseed molasses and 7% cottonseed oil were used as the substrates for SLs production, the titer of SLs was similar to that in the glucose and oleic acid fermentation medium.

### 2.6. Concentration Optimization of Cottonseed Molasses and Cottonseed Oil by Two-Factor Response Surface Analysis

**Figure 1:**
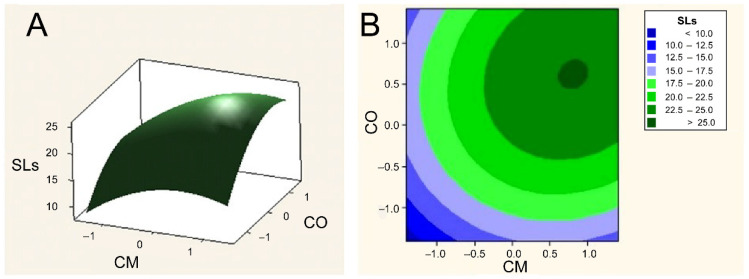
Concentration optimization of cottonseed molasses and cottonseed oil by two-factor response surface method. (**A**) Response surface plot, (**B**)Contour plot.

Through preliminary experiments, with 10% (*w*/*v*) cottonseed molasses and 6% (*v*/*v*) cottonseed oil as the base point, 13 settings were designed for SL production, and the common fermentation medium (CFM) containing glucose and oleic acid was used as the control. Variable combinations and fermentation results were shown in table in Section 3.6, and the results of response surface optimization were shown in Figure 1. It could be seen that when the concentration of cottonseed molasses was 12% (*w*/*v*) and the cottonseed oil concentration was 7% (*v*/*v*), the two-factor response surface experiment had the maximum value. At this time, the titer of SLs was higher than 48.1 g/L, and the production of lactonic SLs was higher than 25.0 g/L. In the meantime, the titer of total SLs and lactonic SLs was 46.2 ± 0.9 g/L and 24.7 ± 1.2 g/L in the control group, respectively. The SL yields produced from cottonseed molasses and cottonseed oil fermentation medium increased by more than 6% compared with what was produced from glucose and oleic acid. In addition, no inorganic salts and nitrogen sources were added to the fermentation media. As a result, the production cost of SLs is further reduced.

From the above optimizations based on the single-factor method and two-factor response surface analysis, the optimal formula of the fermentation medium was 12% (*w*/*v*) cottonseed molasses and 7% (*v*/*v*) cottonseed oil.

### 2.7. Using the Optimal Formula of the Fermentation Medium to Produce SLs

Cottonseed molasses was a waste material to be discarded in the production of raffinose. Based on the above experiments, the fermentation media with cottonseed molasses and cottonseed oil were optimized as follows: 12% (*w*/*v*) cottonseed molasses and 7% (*v*/*v*) cottonseed oil without inorganic malts and yeasts to produce the SLs. In this time, the production of total SLs reached 58.4 ± 3.4 g/L, and lactonic SLs were elevated to more than 25.0 ± 1.9 g/L, which increased by 4.7% and 17.9% compared with SLs production from the medium containing glucose and oleic acid.

The CM and CO fermentation media also save costs by eliminating the need to add inorganic salts and additional nitrogen sources, such as yeast extract. In addition, cottonseed molasses and cottonseed oil were both derived from cottonseed. Cottonseed molasses, a waste material to be discarded in the production of raffinose, can be used as the hydrophilic carbon sources to produce the SLs, which can not only reduce the production cost of SLs but also find a way for its recycling. The price of oleic acid is about twice as high as cottonseed oil. The cost analysis of fermentation media was shown in Tables S3 and S4. The production cost for SL production in CM and CO fermentation media was reduced by 55.9% compared with that in commonly used glucose and oleic acid medium fermentation.

### 2.8. Identification of SLs Components Produced from Glucose Fermentation Medium and Cheap CM Fermentation Medium by HPLC-MS

**Figure 2:**
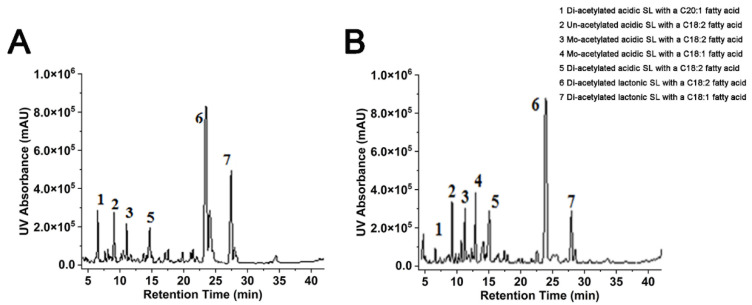
HPLC profiles of SLs produced from glucose fermentation medium and CM fermentation medium by *S. bombicola*. (**A**) HPLC profiles of SLs produced from glucose fermentation medium. (**B**) HPLC profiles of SLs produced from CM fermentation medium. Six main SL components (1, 2, 3, 5, 6, 7) were both produced in the medium with glucose or that with cottonseed molasses, while SL Component 4 can only be produced in the medium with cottonseed molasses.

Figure 2 showed the HPLC profiles of SLs produced from common glucose fermentation medium and cheap CM and CO fermentation medium by *S. bombicola*. There were more than 20 kinds of SL molecules in the two-fermentation media. Six main components were found in the SL mixture from the glucose and oleic medium, while seven components were detected in the SL mixture produced from cheap CM and CO medium.

**Figure 3:**
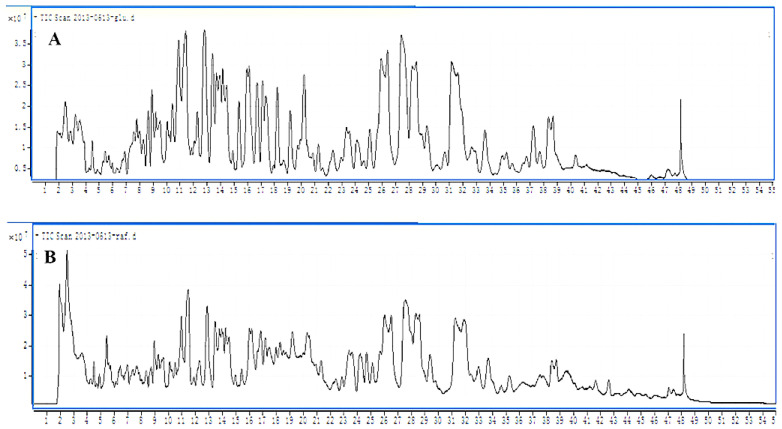
HPLC-MS analysis of SLs component produced from glucose fermentation medium and cheap CM fermentation medium. (**A**) HPLC–Mass spectrometry of SL components produced from glucose fermentation medium. (**B**) HPLC–Mass spectrometry of SL components produced from cheap CM fermentation medium.

HPLC–MS further identified the structures of SLs [24,25], and the results are shown in Figure 3. MS results revealed that the MW of No-1 is 734, corresponding to a di-acetylated acidic SL with a C20:1 DASL. In the CID spectrum of the molecular ion [M + NH_4_]^+^, the protonated ion peak at m/z 735 is obtained by the loss of NH_3_ from m/z 752. The fragment ion peak at m/z 717 is generated by the loss of H_2_O from m/z 735. The loss of AcOC_6_H_9_O_4_ from the protonated ion led to the formation of the ion peak at m/z 531. The fragment ion peak at m/z 513 and 477 resulted from losing one or three H_2_O molecules from m/z 531, respectively. Hydroxy fatty acid loss resulted in fragment ion peak formation at m/z 417, which indicated that the SL molecule has two acetyl groups in sophorose moiety. The ion at m/z 327 (C_20_H_39_O_3_) is the protonated hydroxy fatty acid, and ions at m/z 309(C_20_H_37_O_2_), 291 (C_20_H_35_O), and 273 (C_20_H_33_) originated from m/z 327 by a consecutive loss of three H_2_O molecules, respectively. These four ions indicated that the SL molecule is an acidic SL, and the SL molecule has a 20:1 hydroxy fatty acid. The No-1 is a Di-acetylated acidic SL with a C20:1 fatty acid. Six other sophorolipid molecules are identified by this method presented in Table 5.

The types of SLs produced by glucose and oleic acid medium and CM and CO medium were the same, and CM also produced the six main SLs components produced by glucose. In addition, the main sophorolipid component produced by cottonseed molasses was an acidic sophorolipid molecule with a carbon chain length of 18, a double bond, and a single acetylation (C18:1 SASL). In contrast, the content of this component was shallow in the SLs produced from glucose, and the medium containing cottonseed molasses were more advantageous. In addition, HPLC analysis showed that the peak produced by CM medium, namely C18:2 diacetylated lactonic sophorolipid (C18:2 DLSL), were of higher purity than that by glucose medium. Cottonseed molasses and cottonseed oil are practical alternatives to glucose and rapeseed oil, not only because they are of low cost and do not require the addition of yeast extract and inorganic salts but also because of the high proportion and purity of the component C18:2 DLSL, which was considered to be second only to C18:1 DLSL in terms of biological activity.

## 3. Materials and Methods

### 3.1. Chemicals, Strain, and Media

The chromatographic grade acetonitrile and methanol were all purchased from TEDIA Company Inc. (Fairfield, CT, USA). Anthrone was purchased from Sigma (St. Louis, MI, USA). The antibiotics were purchased from Amresco (Solon, OH, USA). Agar powder, galactose, peptone, sorbitol, and other reagents were of analytical grade and purchased from Dingguo (Beijing, China).

Strain *Starmerella bombicola* CGMCC 1576, an SL-producing yeast strain, was isolated from oil-containing wastewater by our laboratory and conserved in China General Microbiological Culture Collection Center (CGMCC), numbered 1576 [26]. The strain was originally identified as *Wickerhamiella domercqiae var. Sophorolipid* through physiological and biochemical identification tests and was reclassified to be *S. bombicola* based on sequence analysis. The strain *S. bombicola* was used as a wild strain in this study. The strains were all preserved on yeast extract peptone dextrose (YEPD) medium, as described previously [26].

YEPD medium contained (g/L): yeast extract 10.0, peptone 20.0, and glucose 20.0. Glucose fermentation medium contained (g/L): glucose 80.0, oleic acid 6% (*v*/*v*), KH_2_PO_4_ 1.0, Na_2_HPO_4_ 1.0, MgSO_4_·7H_2_O 0.5, yeast extract 3.0, natural pH. Cottonseed molasses (CM) and cottonseed oil (CO) fermentation medium contained: Cottonseed molasses 133.3 g/L, cottonseed oil 6% (*v*/*v*), and natural pH.

### 3.2. Fermentation Media Containing Different Carbon Sources

Six fermentation media were designed with two hydrophilic and three hydrophobic carbon sources. The two hydrophilic carbons include glucose and cottonseed molasses. Cottonseed molasses was analyzed by the method of HPLC. The composition and proportion of the components (%) of cottonseed molasses are as follows: cottonseed sugar 21.08, xylose 0.63, lupeose 4.55, melibiose 1.44, sucrose 25.87, fructose 0.71, glucose 1.35, oligosaccharide 5.17, crude protein 20.20, and water 19.00. When 133.3 g/L of cottonseed molasses (equal to 80.00 g/L glucose) was used as the hydrophilic carbon source, 6% (*v*/*v*) oleic acid, 6% (*v*/*v*) soybean oil, and 6% (*v*/*v*) cottonseed oil were used as hydrophobic carbon sources, respectively. Meanwhile, when 80 g/L of glucose was used as the hydrophilic carbon source, the hydrophobic carbon sources used were the same as those used for glucose media. The pH of all media is natural. After seven days of cultivation at 200 rpm and 25 °C, the biomass of S. bombicola, lactonic SLs, and total SL production was measured.

### 3.3. CM and CO Fermentation Media Containing Different Concentrations of Yeast Extract

Cottonseed molasses contains a high crude protein content, which can probably be used as the nitrogen source for the fermentation of *S. bombicola*. To determine the effect of yeast extract on the production of SLs, *S. bombicola* was grown in CM and CO media containing different concentrations of yeast extract (g/L): 0.0, 1.0, 2.0, 3.0, and 4.0, respectively. After seven days of cultivation at 200 rpm and 25 °C, the biomass of *S. bombicola*, lactonic SLs, and total SL production was measured.

### 3.4. CM and CO Fermentation Media with or without Inorganic Salts

Cottonseed molasses has a high content of inorganic salts, so *S. bombicola* was maybe grown in the CM and CO medium without the need for the addition of inorganic salts. We redesigned CM and CO fermentation media, with one medium containing inorganic salts (g/L): KH_2_PO_4_ 1.0, Na_2_HPO_4_ 1.0, and MgSO_4_·7H_2_O 0.5. The other had no inorganic salt ions. Cottonseed molasses 133.3 and cottonseed oil 6% (*v*/*v*) were added to both media, natural pH. After seven days of cultivation at 200 rpm and 25 °C, the biomass of *S. bombicola*, lactonic SLs, and total SL production was measured.

### 3.5. Optimization of CM and CO Concentrations by Single-Factor Experiment

Two groups of fermentation media were prepared. In one group, the concentrations of cottonseed molasses (*w*/*v*) were 8.3%, 10.0%, 11.7%, 13.3%, and 15.0%, respectively, and the concentration of cottonseed oil was set at 6% (*v*/*v*). The other group set molasses at 11.7% (*w*/*v*), and the concentrations of cottonseed oil (*v*/*v*) were set at 5.0%, 6.0%, 7.0%, 8.0%, and 9.0% pH natural, respectively. After 7 days of cultivation at 200 rpm and 25 °C, the biomass of *S. bombicola*, lactonic SLs, and total SLs production was measured.

### 3.6. Optimization of Fermentation Medium Containing CM and CO by Two-Factor Response Surface Method

The concentrations of cottonseed molasses and cottonseed oil were considered as two factors, with 10% (*w*/*v*) cottonseed molasses and 6% (*v*/*v*) cottonseed oil as the base point. Thirteen groups of experiments were designed with 2% as a unit of change. The experiment design was shown in Table 6. The seed culture was inoculated into fermentation medium (50 mL in 300 flask) at an inoculum of 2% (*v*/*v*). After 7 days of cultivation at 200 rpm, 25 °C, the biomass of *S. bombicola*, lactonic SLs, and total SLs production was measured.

### 3.7. Determination of Residual Glucose, Biomass, SLs Production

Five mL of culture broth (in triplicate) of *S. bombicola* were mixed with n-butanol/ethanol/chloroform (10/10/1) of equal volume and then centrifuged. After being washed twice in distilled water, the solid residue was dried and weighed. Biomass was measured by dry weight method, and the data of biomass were presented as the mean of three readings. The titer of lactonic SLs was measured by extraction of 0.5-mL fermentation broth with two volumes of ethyl acetate and, finally, using the anthrone method [23]. For determination of total SL titer, two volumes of acetonitrile were added to 0.5-mL broth to dissolve SLs and then centrifugated at 8000 rpm for 10 min. Total SL production was calculated according to the glucose standard curve with glucose content of total sugar content, minus residual glucose content. For both lactonic and total SL production determinations, 10 μL of the samples were taken out in duplicate, followed by reacting with anthrone solution. Finally, 200 μL of the reaction mixture were taken out in triplicate and determined at OD_620_. The data were presented as the mean of six readings.

### 3.8. Composition Analysis of SLs by HPLC and HPLC-MS

The composition of crude total SLs was analyzed by analytical HPLC (Shimadzu, Japan) with a Venusil MP–C18 column (250 × 4.6 mm, Bonna–Agela Technologies Inc., Torrance, CA, USA) and a UV detector at 207 nm. Acetonitrile/water was used as the mobile phase at an acetonitrile gradient from 40% to 60% for 15 min followed by an acetonitrile gradient from 60% to 70% for 35 min at a flow rate of 1.0 mL/min. The data of HPLC-mass spectrometry (HPLC-MS) were presented as the mean of three independent experimental results with the same injection volume.

API 4000 mass spectrometer (Applied Biosystems, Foster City, CA, USA) was used to perform MS analysis on each peak of HPLC.

## 4. Conclusions

In this study, cottonseed molasses and cottonseed oil were used as hydrophilic and hydrophobic carbon sources to ferment SLs. Nitrogen sources and inorganic salts were optimized successively. The optimization of CM and CO cheap medium by the single-factor method and two-factor response surface analysis were performed to determine the optimal concentrations of cottonseed molasses and cottonseed oil. The optimal carbon sources were 12% cottonseed molasses and 7% cottonseed oil after repeated verification. The SL components produced by fermentation medium with glucose and fermentation medium with cottonseed molasses were identified and analyzed by HPLC–MS. *S. bombicola* could produce SLs by using cottonseed molasses and cottonseed oil, and the two cheap substrates are advantageous for the production of SLs because they are waste from the process of raffinose and do not require the addition of yeast extract powder and inorganic salts.

China is the world’s largest cotton producer; cottonseed resources are abundant. How to effectively use many valuable components in cottonseed to increase the total profit rate has aroused the attention of manufacturers and scientific research institutions. Therefore, using cottonseed oil and cottonseed molasses as the carbon sources to produce SLs can expand the utilization of cottonseed. Moreover, at the same time, it provides the possibility for the large-scale industrial production of SLs.

## Figures and Tables

**Table 1:** Chemical compositions of different hydrophobic carbon sources.

Hydrophobic Carbon Sources	Palmitic Acid (%)	Stearic Acid (%)	Oleic Acid (%)	Linoleic Acid (%)	Linolenic Adid (%)	Arachidonic Acid (%)
Oleic acid	-	-	100	-	-	-
Soybean oil	7.0 ± 1.0	4.0 ± 1.0	30.5 ± 5.5	58.5 ± 6.5	2.5 ± 0.5	0.3 ± 0.2
Cottonseed oil	23.2 ± 1.6	2.2 ± 0.3	24.4 ± 6.4	50.0 ± 5.1	-	0.1 ± 0.1

**Table 2:** Fermentation results of *S. bombicola* using different carbon sources.

Hydrophilic Carbon Source	Hydropobic Carbon Source	Total SLs (g/L)	Lactonic SLs (g/L)	Biomass (g/L)
Glucose	Oleic acid	55.8 ± 2.8	21.1 ± 3.4	7.2 ± 0.3
	Soybean oil	63.7 ± 0.5	24.1 ± 3.1	6.3 ± 0.5
	Cottonseed oil	72.4 ± 2.3	24.5 ± 1.8	7.3 ± 0.8
Cottonseed molasses	Oleic acid	53.2 ± 1.7	18.9 ± 0.9	9.5 ± 0.6
	Soybean oil	61.7 ± 2.6	19.3 ± 2.4	10.0 ± 0.6
	Cottonseed oil	68.9 ± 4.9	21.2 ± 1.6	8.8 ± 0.4

**Table 3:** Fermentation of *S. Bombicola* in CM and CO media containing different concentrations of yeast extract.

Carbon Source	Yeast Extract (%)	Total SLs (g/L)	Lactonic SLs (g/L)	Biomass (g/L)
Glu + oleic acid	0.3	50.0 ± 3.7	28.2 ± 1.7	9.0 ± 1.2
CM + CO	-	49.2 ± 2.1	27.6 ± 2.4	7.3 ± 0.6
	0.1	43.3 ± 1.6	22.1 ± 0.8	12.8 ± 1.3
	0.2	42.9 ± 3.4	22.0 ± 1.5	12.8 ± 0.5
	0.3	42.1 ± 1.7	21.2 ± 0.9	13.2 ± 0.9
	0.4	41.5 ± 2.3	20.8 ± 3.8	13.9 ± 1.6

Note: CM is short for cottonseed molasses; CO is short for cottonseed oil.

**Table 4:** Effects of inorganic salts on the production of SLs of *S. bombicola*.

Carbon Source	KH_2_PO_4_ (g/L)	Na_2_HPO_4_ (g/L)	MgSO_4_·7H_2_O (g/L)	total SLs (g/L)	Lactonic SLs (g/L)	Biomass (g/L)
CM + CO	1.0	1.0	0.5	60.8 ± 2.2	24.0 ± 3.1	6.0 ± 0.4
	-	-	-	57.6 ± 2.3	24.0 ± 1.2	6.7 ± 0.7

Note: CM is short for cottonseed molasses; CO is short for cottonseed oil.

**Table 5:** Molecular weight, structure elimination, and abbreviation of seven main sophorolipid components detected in both glucose and CM fermentation media.

No	MW	Sophorolipid Molecule	Abbreviation
1	734	Di-acetylated acidic SL with a C20:1 fatty acid	C20:1, 2AC, acidic SL
2	620	Un-acetylated acidic SL with a C18:2 fatty acid	C18:2, 0AC, acidic SL
3	662	Mo-acetylated acidic SL with a C18:2 fatty acid	C18:2, 1AC, acidic SL
4	664	Mo-acetylated acidic SL with a C18:1 fatty acid	C18:1, 1AC, acidic SL
5	704	Di-acetylated acidic SL with a C18:2 fatty acid	C18:2, 2AC, acidic SL
6	686	Di-acetylated lactonic SL with a C18:2 fatty acid	C18:2, 2AC, lactonic SL
7	688	Di-acetylated lactonic SL with a C18:1 fatty acid	C18:1, 2AC, lactonic SL

**Table 6:** Experimental design and results of response surface optimization.

Standard No.	Operation No.	PtType	Block	CM	CO	Lac SLs (g/L)
3	1	1	1	−1	1	23.70
9	2	0	1	0	0	23.97
2	3	1	1	1	−1	21.36
7	4	−1	1	0	−1.41	23.52
10	5	0	1	0	0	23.97
4	6	1	1	1	1	23.77
5	7	−1	1	−1.41	0	24.28
11	8	0	1	0	0	23.97
13	9	0	1	0	0	23.97
1	10	1	1	−1	−1	23.59
6	11	−1	1	1.41	0	23.89
8	12	−1	1	0	1.41	23.75
12	13	0	1	0	0	23.97

Note: CM is short for cottonseed molasses; CO is short for cottonseed oil.

## Data Availability

All data generated or analysed during this study are included in this article and its supplementary information files.

## Supplementary Materials

The following supporting information can be downloaded at: https://www.mdpi.com/article/10.3390/ijms24065759/s1.
