# Supplementary material for: Reduced-Cost Production of Sophorolipids by Starmerella bombicola CGMCC1576 Grown on Cottonseed Molasses and Cottonseed Oil-Based Medium
Source: Int J Mol Sci. 2023 Mar 17;24(6):5759. doi: 10.3390/ijms24065759 (PMC10057841; doi:10.3390/ijms24065759)
Supplement: Supplementary file 1 [file ijms-24-05759-s001.zip › ijms-2236321-supplementary.pdf]

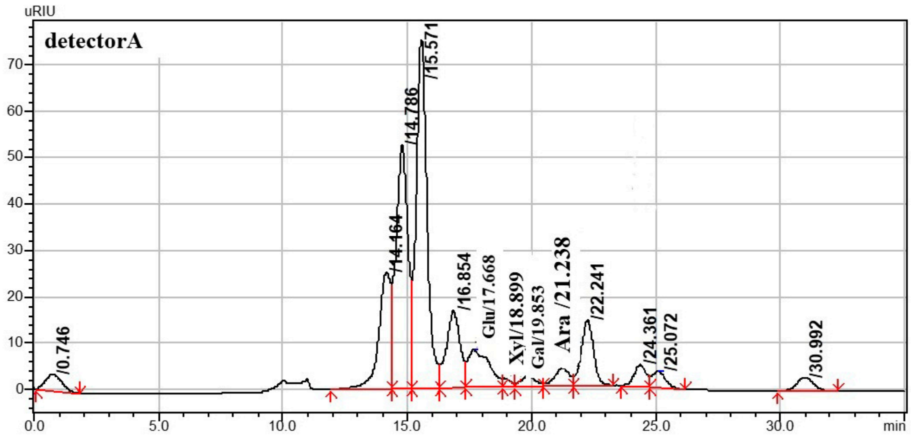

Figure S1. Composition analysis of cottonseed molasses by HPLC.

Table S1. Concentration optimization of cottonseed molasses.

Carbon Source	Hydrophilic Carbon Source (%)	Total SLs (g/L)	Lac SLs (g/L)	Biomass (g/L)
Glu + oleic acid	8.0	50.5 ± 3.8	28.0 ± 1.6	9.0 ± 0.3
	8.3	44.8 ± 2.1	21.8 ± 1.6	13.0 ± 0.9
	10.0	46.2 ± 3.0	22.6 ± 0.9	13.0 ± 0.1
CM + CO	11.7	48.9 ± 1.3	24.5 ± 1.1	12.3 ± 0.6
	13.3	43.2 ± 1.9	21.0 ± 0.5	13.2 ± 0.2
	15.0	41.1 ± 2.5	19.4 ± 1.3	14.5 ± 0.7

Note: CM is short for cottonseed molasses; CO is short for cottonseed oil.

Table S2. Concentration optimization of cottonseed oil.

carbon source	Hydrophobic Carbon Souce (%)	Total SLs (g/L)	Lac SLs (g/L)	Biomass (g/L)
Glu + oleic acid	6.0	50.5 ± 1.5	28.2 ± 0.7	9.2 ± 0.3
	5.0	46.2 ± 0.9	24.7 ± 1.2	6.3 ± 0.2
	6.0	49.1 ± 1.1	27.0 ± 0.8	8.5 ± 0.3
CM + CO	7.0	50.6 ± 1.8	27.7 ± 0.3	8.9 ± 0.5
	8.0	46.3 ± 0.3	25.4 ± 0.4	7.8 ± 0.1
	9.0	42.7 ± 1.0	23.9 ± 0.4	7.3 ± 0.5

Note: CM is short for cottonseed molasses; CO is short for cottonseed oil.

Table S3. Cost analysis of commonly used fermentation medium.

Ingredient	Price ($/ton)	Content (%)	Price ($/ton)
glucose	574	8.0	45.92
yeast extract	15781	0.3	47.34
oleic acid	1735	6.0	104.10
KH_2PO_4	7061	0.1	7.06
Na_2HPO_4		0.1	1.07
$MgSO_4·7H_2O$	1449	0.05	0.73

Total Price	-	-	206.22

Table S4. Cost analysis of fermentation medium with CM and CO.

Ingredient	Price ($/ton)	Content (%)	Price ($/ton)
Cottonseed oil	780	7.0	54.6
Cottonseed molasses	0	12.0	0
Total price	-	-	54.6

Note: CM is short for cottonseed molasses; CO is short for cottonseed oil.
